# Incidence of bla genes among uropathogenic *Escherichia coli* isolates from HIV and non-HIV patients in South India

**DOI:** 10.1186/1471-2334-12-S1-P21

**Published:** 2012-05-04

**Authors:** Kesavaram Padmavathy, Padma Krishnan, Sikhamani Rajasekaran

**Affiliations:** 1 Dept of Microbiology, Dr. ALM PGIBMS, University of Madras, Chennai, India; 2 Sree Balaji Dental College and Hospital, Chennai, India; 3 Government Hospital of Thoracic Medicine, Chennai, India

## Background

Group 3a/b cephalosporins are currently being used in the treatment of UTI and urosepsis. However, Extended Spectrum Beta-Lactamase (ESBL) mediated resistance has been increasingly reported among uropathogens from HIV patients. We sought to determine the incidence of ESBL genes- *bla_CTX_*_-M_, *bla_TEM_* and *bla_SHV_* among *E. coli* isolates from HIV (with increased exposure to cephalosporins) and non-HIV antenatal patients.

## Methods

PCR detection of *bla_CTX-M_*, *bla_TEM_* and *bla_SHV_* were carried out among ESBL producing urinary *E. coli* isolates from HIV (n=57) and non-HIV antenatal patients (n=22). Fisher’s exact test was employed to analyze the statistical significance of the results.

## Results

Overall, 31.7%, 59.5% of the *E. coli* isolates carried *bla_TEM_*, *bla_CTX-M_* respectively, while none harboured bla_SHV_. When stratified based on host group, significant difference was observed in the incidence of *bla_CTX-M_* among the isolates from HIV and non-HIV patients (70.2% vs 31.8% respectively, p = 0.0024; OR 5.042; 95% CI = 1.7441-14.5759). Nonetheless, difference in prevalence of *bla_TEM_* among the HIV and non-HIV isolates was not statistically significant (29.8% vs 36.4%, p = 0.5979). Co-occurrence of *bla_TEM_* and *bla_CTX-M_* was detected among 22.8%, 0% of the *E. coli* isolates from HIV and non-HIV patients respectively (OR 5.1447; 95% CI = 1.3766-19.2273).

## Conclusion

Our results augment the fact that frequent exposure to cephalosporins serves as the driving selection force leading to increased incidence of ESBL (*bla_CTX-M_*) mediated resistance among the *E. coli* isolates from HIV patients. Hence, the risk associated with antimicrobial exposure needs to be considered in therapeutic decision making.

